# Efficacy of an Immune Modulator in Experimental *Chlamydia trachomatis* Infection of the Female Genital Tract

**DOI:** 10.1155/IDOG/2006/61265

**Published:** 2006-03-07

**Authors:** Joseph M. Lyons, James I. Ito, Servaas A. Morré

**Affiliations:** Department of Infectious Diseases, City of Hope National Medical Center and Beckman Research Institute, Duarte, CA 91010, USA; Laboratory of Immunogenetics, Section Immunogenetics of Infectious Diseases, VU University Medical Center, 1007 MB Amsterdam, The Netherlands

## Abstract

*Objective*. The aim of this study was to determine if vaginal application of the immune response modifier imiquimod (Aldara cream, 3M Pharmaceuticals, St Paul, Minn) would alter the course and/or outcome of female genital tract infection with a human isolate of *Chlamydia trachomatis* in a murine model. *Methods*. Groups of CF-1 mice were treated with Aldara on three different schedules: (1) ongoing beginning 5 days prior to and continuing through day 5 of infection; (2) a single prophylactic dose 2 hours prior to infection; and (3) therapeutic from day 4 to day 14 of infection. Mice were infected vaginally with a serovar D strain of *C trachomatis*, and monitored by culture to determine the level of shedding and duration of infection. *Results*. We observed a significant reduction in both duration of infection and the level of shedding during the acute phase in mice treated on an ongoing basis commencing 5 days prior to infection. There was no effect with respect to the other regimens. *Conclusion*. These results demonstrate that ongoing Aldara treatment has efficacy and may enhance local innate immunity which reduces the duration of subsequent infection with human isolates of *C trachomatis* in a murine model of female genital tract infection.

## INTRODUCTION

Imiquimod is an immune response modifier [1] that under the trade name of Aldara (3M Pharmaceuticals, St Paul, Minn) has FDA approval for the external treatment of anogenital warts. In addition to this highly effective application [2], imiquimod has been shown to be effective in an increasing number of dermatological conditions, including common warts, actinic keratoses, and basal cell carcinomas, as well as lentigo malina and molluscum contagiosum [3]. In two other infectious diseases with cutaneous manifestations, genital herpes and leishmaniasis, it has demonstrated significant efficacy in animal models [4, 5], although results in humans have been equivocal [6–9]. In all of these settings, imiquimod is thought to act by binding independently to toll-like receptors (TLR)-7 and TLR-8 and activating the signal transduction cascade that triggers transcription factor NK-kB, which in turn upregulates production of cytokines and chemokines that direct a sustained T_H_1 dominant cellular immune response [10–12]. Both TLR-7 and TLR-8 have been shown to be expressed on both human and mouse cells [13], making mouse models of human disease useful tools in the assessment of the treatment potential of imiquimod in those settings where such models exist.

*Chlamydia trachomatis* is the most prevalent sexually transmitted bacterial pathogen in the world, with an estimated 89 million new cases occurring each year [14]. The significant morbidity associated with the severe sequelae of *C trachomatis* genital tract infection in women has driven efforts to both control the spread of and to effectively treat those infected with this human pathogen. Although highly effective antibiotics are available [15, 16], the incidence has been on the increase since the mid 1990s [14], and despite considerable efforts over the last 20 years, no vaccine candidates have emerged. It is generally accepted that the immune responses made during persistent and recurrent infections contribute to the immunopathic nature of the severe sequelae, which includes pelvic inflammatory disease, tubal infertility, and ectopic pregnancy [17–20]. While on the other hand, T_H_1 dominant immune responses have been shown to play a role in controlling the spread of infection within the genital tract and to provide some level of acquired immunity as evidenced by reduced shedding of infectious units and shorter duration of infection following reinfection [21]. Taken together, these facts make the use of immune modulators that enhance local T_H_1 dominant responses an appealing approach to prophylacticly and/or therapeutically treat *C trachomatis* female genital tract infection.

Much of our understanding of *C trachomatis* female genital tract infection has been derived from the use of a murine model of noninvasive lower genital tract infection developed by Tuffrey and Taylor-Robinson [22]. In our laboratory, we use this model and routinely infect mice with chlamydial strains belonging to the oculogenital biovar of *C trachomatis* (serovars D-K), which results in an infection that in most of its features closely mimics human infection, both in its course and outcome [23]. Using this model, we assessed the efficacy of imiquimod to alter the course of infection under three different vaginal application schedules: (1) ongoing beginning 5 days prior to and continuing through day 5 of infection; (2) a single prophylactic dose 2 hours prior to infection; and (3) therapeutic from day 4 to day 14 of infection. Efficacy was evaluated by comparing the susceptibility to infection, the level of shedding, and the duration of infection between imiquimod- and placebo-treated groups of mice.

## MATERIALS AND METHODS

### Murine model

Female CF-1 mice were purchased from Charles River Laboratories (Wilmigton, Mass) and were used at 7-8 weeks of age. In order to interrupt the normal 4-5 day estrous cycle and induce prolonged diestrous and thus enhance the initial infection rate, progesterone in the form of medroxyprogesterone acetate (Depo-Proveva, Pharmacia & Upjohn Co Peapeck, NJ), was administered subcutaneously in 2.5 mg doses, 10 and 3 days prior to infection [22, 23]. Mice were inoculated intravaginally by direct instillation of 10 μL of *C trachomatis* (serovar D) bacterial suspension containing 1 × 10^5^ inclusion forming units (IFU). The human *C trachomatis* serovar D genital isolate was propagated, titrated, and isolated in cycloheximide-treated McCoy cell monolayers using standard techniques [24]. All experiments were conducted in a BL-2 containment facility in compliance with animal care regulations and under protocols approved by the Institutional Research Animal Care Committee.

### Imiquimod treatment

On the days prior to (−) and/or after (+) infection as indicated in the results table, 10 uL of Aldara diluted 1 : 4 in saline or a placebo similar in composition to the inactive base used in Aldara was administered intravaginally. The following Aldara regimens were tested: (1) Aldara or base at days −5, −3, −1, +1, +3, +5; (2) Aldara or base at days +4, +6, +8, +10, +12, +14; (3) Aldara or base 2 hours prior to infection; and (4) neither Aldara nor base.

### Infection monitoring

The presence of *Chlamydia* in the lower genital tract was determined by culturing the material obtained by swabbing the vaginal vault and ectocervix with a dacron-tipped swab that was stored at −70°C in 2-SPA transport medium until tested. Specimens were plated onto McCoy monolayers in duplicate microtiter plates, centrifuged, and incubated at 37°C for 72 hours. One plate was then fixed, stained with iodine and inclusions were enumerated (actual IFU counts in table), while the other plate was stored at −70°C and used to verify the status of primary culture negative specimens. A specimen was considered culture positive if inclusions were observed in either primary or secondary culture.

### Statistic analysis

The duration of infection between groups was analyzed using the Wilcoxon rank sum test.

## RESULTS

As displayed in Table 1, we observed a statistically significant reduction in the median duration of infection in mice treated on multiple occasions prior to and during the acute phase of *C trachomatis* infection, 4 days for imiquimod-treated animals as compared to 19 days for the placebo group (*P* < 0.5). Remarkably, although no effect was observed on the incidence of infection, there was a suggestion that the number of IFU shed during the first round of intracellular replication (day 2) was reduced when compared to the placebo group. This is especially marked when the average number of IFU shed by treated mice with a duration of infection less than or equal to the median duration of the group is compared to the average number of IFU shed by the placebo group on day 2, 224 IFU versus 2010 IFU, respectively. By day 4 the differences between the ongoing treatment group and placebo group were unequivocal, with 6 of 8 treated mice being culture negative in primary culture while all four placebo control mice were primary culture positive.

Following the discontinuation of imiquimod treatment on day 5 postinfection, 5 of 8 treated mice were culture negative for the duration of the study period, while the remaining 3 mice in this group had shedding patterns and durations of infection similar to placebo mice.

The other regimens assessed, a single prophylactic application 2 hours prior to infection (data not shown) and a 6-application therapeutic regimen commencing on day 4 postinfection, had neither a positive nor negative effect on either the duration of infection or the pattern of shedding during infection.

## DISCUSSION

Ramsey and colleagues [25] recently reported in this journal that imiquimod had no efficacy in modifying the susceptibility to or course of infection with the mouse pneumonitis biovar (MoPn) of *Chlamydia* when administered either orally or intravaginally in essentially the same murine model of *C trachomatis* female genital tract infection used in the present study. In that study, imiquimod was administered to groups of inbred BALB/c mice either orally on five occasions (3 days prior to infection, on the day of infection, and 1, 3, and 6 days after infection), or intravaginally on four occasions (2 days prior to infection, and 1, 3, and 6 days after infection). These authors concluded that although the results do not hold promise for imiquimod in therapy for chlamydial infection, the efficacy of imiquimod may have been masked due to the obvious potent T_H_1 response that naturally occurs during genital tract infection with MoPn and/or that the TLR-7 expression was absent or insufficient within the gastrointestinal and genital tracts to allow a response to imiquimod. We, on the other hand, observed a significant reduction in the median duration of infection and level of shedding in outbred CF-1 mice treated intravaginally on six occasions (5, 3, and 1 days prior to infection and 1, 3, and 5 days after infection) with a strain belonging to the oculogenital biovar of *C trachomatis*, the biovar that actually causes human disease. Based on this result, we would conclude that TLR-7 and/or TLR-8 is expressed in the female mouse genital tract, and that imiquimod and/or immune modulators as a class might have some, albeit limited, place in the therapy of chlamydial infection. In a letter to the Editor, we questioned the utility of MoPn to serve as a surrogate for the oculogenital biovar and referenced our finding [26], the details of which form the basis of this report.

Although differences in the details of the two studies, such as mouse strain and intravaginal application schedule, could explain the contrary results obtained in these two studies, in our opinion the major difference in experimental design that contributed to the different outcomes was the selection of the biovar of *Chlamydia* against which to assess the efficacy of this potent immunomodulator. Multiple phenotypic differences that bear directly on the ability to detect the potential infection and immunity altering effects of imiquimod have been reported between *C muridarum*, MoPn, and the 11 different serovars that comprise the human oculogenital biovar of *C trachomatis* [27]. Most notable among these differences are those that arise from variability in both the unique obligate intracellular developmental cycle [28] and the degree of tryptophan auxotrophy [29] that exists within the genus *Chlamydia*. Taken together, phenotypes associated with these two characteristics of a given strain define the extent to which interferon-gamma can exert a nutritional influence on the chlamydial replication cycle via tryptophan depletion following IDO induction. In the present context, MoPn has been shown to be significantly less sensitive to the tryptophan depleting effect of interferon-gamma when compared in vitro to strains belonging to different serovars of the oculogenital biovar, including a strain identified as serovar D [30]. In addition, the course and outcome of MoPn infection in the female mouse genital tract is essentially independent of interferon-gamma compared to infection with serovar D, as evidenced by uncontrolled and invasive progression of disease during serovar D infection in interferon-gamma deficient mice compared to the controlled and contained course seen in interferon-gamma sufficient mice [31, 32]. This latter difference between the biovars is likely to be a result of a mechanism that involves basic elements of the T_H_1 innate response that are induced by interferon-gamma, including events triggered in the host cell by IDO-mediated tryptophan depletion [33, 34], as well as events associated with the significantly more rapid replication and release kinetics characteristic of the MoPn developmental cycle [28]. This latter phenotypic difference might favor the escape of MoPn from many of the immediate consequences of the potent T_H_1 responses that it induces, effects that cannot be escaped by the slower replicating strains of the oculogenital biovar.

Imiquimod is thought to exercise its antiviral, antitumor, and antimicrobial activity through multiple pathways that promote a T_H_1 dominant response that augments the cell-mediated immune responses of both the innate and acquired immune systems. This is achieved through the induction, via TLR-7 and/or TLR-8, of an array of immune response modifiers, most notably interferon-γ and tumor necrosis factor-α, as well as interferon-α, G-CSF, GM-CSF, the interleukins, IL-1, IL-2, IL-6, IL-8, and IL-12, and chemokines such as MIP-1a, MIP-1b, and MCP-1 [1, 35]. In addition, imiquimod has been shown to induce nitric oxide synthase, enhance antigen presentation by dendritic cells [36], and most recently to induce apoptosis [37]. Many of these elements of innate and adaptive immunity have been independently assessed in the murine model used in this study, and some have been shown to play a role in the protective immunity that follows infection [38]. However, much of this knowledge has been obtained using MoPn as a surrogate model agent for the oculogenital biovar that actually causes human disease. Unfortunately, the translational value of much of this work may be in question as a result of numerous differences that have been recently reported between MoPn and the human biovar [39]. Two of these differences were drawn upon to help explain the discrepancy between our results and those of Ramsey and colleagues, and other differences might also play a role.

In conclusion, using the murine model of human female genital tract infection and in contrast to the results of Ramsey and colleagues, we observed that intravaginally applied imiquimod significantly reduced the median duration of genital tract infection with a human isolate of *C trachomatis*. When administered on multiple occasions, days prior to and during the acute phase of infection, both the duration of infection and the level of shedding in some mice were reduced, perhaps through a mechanism of enhanced local innate immunity. Understanding the mechanism of this enhanced responsiveness might provide insight into the complex immunobiology of female genital tract infection with *C trachomatis* and may lead to new prevention and treatment methods. Also, given that acquired protective immunity to *C trachomatis* shares many elements in common with the innate immune responses made during initial exposure [33], one might speculate that imiquimod could be effective in previously infected women by promoting an enhanced anemnestic response to reinfection. It is also interesting to speculate if women using the multiple application regimen of imiquimod approved for the treatment of genital warts might realize an alteration in the cytokine responsiveness within the genital tract and a possible reduced risk of *C trachomatis* genital tract infection.

## Figures and Tables

**Table 1:** The influence of multiple intravaginal application regimens on the course of *C trachomatis* genital tract infection.

Treatment	Animal	Culture results on indicated day postinfection^*^																Duration	Median	Rank sum
group		2	4	7	10	14	17	21	24	28	35	42	49	53	60	63	67		duration	*P* value

Prophylactic imiquimod −5, −3, −1, +1, +3, +5	A3	170	+	−	−	−	−	−	−	−	−	−	−	−	−	−	−	4
	A4	10	−	540	3470	490	+	−	−	−	−	−	−	−	−	−	−	17
	A5	310	+	−	−	−	−	−	−	−	−	−	−	−	−	−	−	4
	A6	140	−	−	−	−	−	−	−	−	−	−	−	−	−	−	−	2	4	.05
	A15	100	−	−	−	−	−	−	−	−	−	−	−	−	−	−	−	2		
	A16	11 380	+	1060	7570	−	−	−	−	−	−	−	−	−	−	−	−	10
	A25	8610	990	1350	10	10	+	+	+	+	−	−	−	−	−	−	−	28
	A0	400	10	−	−	−	−	−	−	−	−	−	−	−	−	−	−	4
Placebo −5, −3, −1, +1, +3, +5	B1	870	380	15 540	2370	+	+	+	+	+	+	+	+	−	+	+	−	63
	B2	4980	300	1130	−	−	−	−	−	−	−	−	−	−	−	−	−	7	19	—
	B3	1460	5490	32 020	4850	50	+	+	+	+	−	−	−	−	−	−	−	28		
	B4	730	1670	23 610	780	−	−	−	−	−	−	−	−	−	−	−	−	10
	E1	20	370	33 590	90	10	+	+	−	−	−	−	−	−	−	−	−	21
	E2	400	610	1210	10	+	+	+	+	+	+	−	−	−	−	−	−	35
Therapeutic	E3	240	1030	5260	40	−	−	−	−	−	−	−	−	−	−	−	−	10
imiquimod	E4	1390	4740	2430	470	−	+	+	+	+	+	−	−	−	−	−	−	35	35	Not significant
+4, +6, +8	E5	160	50	20	+	+	+	+	+	+	+	−	−	−	−	−	−	35
+10, +12, +14	E6	270	220	8960	+	+	+	+	−	−	+	+	−	−	−	−	−	42
	E15	320	250	260	−	−	−	−	−	−	−	−	−	−	−	−	−	7
	E0	4970	500	610	−	−	+	+	+	+	+	−	−	−	−	−	−	35
Placebo +4, +6, +8, +10, +12, +14	F2	320	10	60	30	−	−	−	−	−	−	−	−	−	−	−	−	10
	F4	8840	860	210	20	+	+	+	+	−	−	−	−	−	−	−	−	24	26	—
	F16	1270	1040	612	60	+	+	+	+	+	+	+	−	−	−	−	−	42
	F25	760	1730	19 660	70	200	+	+	+	+	−	−	−	−	−	−	−	28
None	G1	+	−	−	−	−	−	−	−	−	−	−	−	−	−	−	−	2
	G2	1170	1290	990	+	+	+	+	+	+	+	+	+	+	−	−	−	53
	G3	30	10	11 040	10	−	−	−	−	+	−	−	−	−	−	−	−	28
	G4	260	460	6010	20	−	−	−	−	−	−	−	−	−	−	−	−	10	28	^**^
	G5	700	610	4280	+	30	−	+	+	+	−	−	−	−	−	−	−	28		
	G6	3350	5140	2194	−	−	−	+	−	−	−	+	−	−	−	−	−	42		
	G14	810	420	341	10	−	−	−	−	−	−	−	−	−	−	−	−	10		
	G25	1620	340	83	10	−	−	−	+	−	+	+	−	−	−	−	−	42		

*Mice were sampled every 2–7 days postinfection. Numerical values represent the number of IFU enumerated in primary culture through day 14; +/− represents the presence (+) or absence (−) of inclusions upon secondary subculture.

**Not different from either placebo group.
